# Real-world evaluation of a QCM-based biosensor for exhaled air

**DOI:** 10.1007/s00216-024-05407-5

**Published:** 2024-06-26

**Authors:** Augusto Juste-Dolz, William Teixeira, Yeray Pallás-Tamarit, Mario Carballido-Fernández, Javier Carrascosa, Ángela Morán-Porcar, María Ángeles Redón-Badenas, María Gracia Pla-Roses, María Dolores Tirado-Balaguer, María José Remolar-Quintana, Jon Ortiz-Carrera, Ethel Ibañez-Echevarría, Angel Maquieira, David Giménez-Romero

**Affiliations:** 1 Instituto Interuniversitario de Investigación de Reconocimiento Molecular y Desarrollo Tecnológico (IDM), Universitat Politècnica de València, Universitat de València, Camino de Vera s/n, 46022 Valencia, Spain; 2 Hospital General Universitario de Castellón, Avinguda de Benicàssim, 128, 12004 Castellón de la Plana, Spain; 3 Universidad CEU Cardenal Herrera, Calle Grecia, 31, 12006 Castellón de la Plana, Spain; 4 La Fe University and Polytechnic Hospital, Avinguda de Fernando Abril Martorell, nº 106, 46026 Valencia, Spain; 5 Departamento de Química, Universitat Politècnica de València, Camino de Vera s/n, 46022 Valencia, Spain; 6 Departamento de Química-Física, Universitat de València, Calle Doctor Moliner 50, 46100 Burjassot, Spain

**Keywords:** Virusmeter, Breathalyzer, QCM, Diagnosis

## Abstract

The biosensor, named “virusmeter” in this study, integrates quartz crystal microbalance technology with an immune-functionalized chip to distinguish between symptomatic patients with respiratory diseases and healthy individuals by analyzing exhaled air samples. Renowned for its compact design, rapidity, and noninvasive nature, this device yields results within a 5-min timeframe. Evaluated under controlled conditions with 54 hospitalized symptomatic COVID-19 patients and 128 control subjects, the biosensor demonstrated good overall sensitivity (98.15%, 95% CI 90.1–100.0) and specificity (96.87%, 95% CI 92.2–99.1). This proof-of-concept presents an innovative approach with significant potential for leveraging piezoelectric sensors to diagnose respiratory diseases.

**Graphical abstract:**

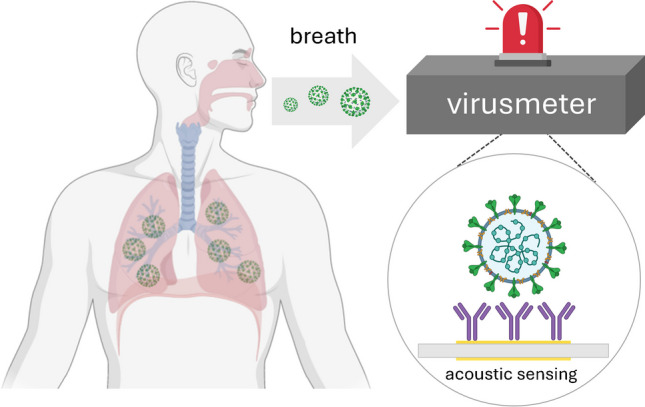

**Supplementary Information:**

The online version contains supplementary material available at 10.1007/s00216-024-05407-5.

## Introduction

Airborne transmission of pathogens, prominently evident in the global coronavirus disease of 2019 (COVID-19) pandemic [1], stands as a crucial driver of respiratory virus dissemination within communities [2, 3]. Inhalation represents the primary route of exposure to viral aerosols, ranging in size of particles from 0.3 to 10 μm upon exhalation. This mode of transmission includes both short-range (< 1 m) and long-range modes, presenting significant challenges in epidemic prevention and control [4–6]. Historical pandemics such as the 2003 SARS pneumonia and the 2009 influenza A (H1N1) also experienced airborne transmission [7].

Research has established a notable link between specific volatile organic compounds (VOCs) and respiratory illnesses [8], emphasizing the need to rethink diagnostic methods for respiratory conditions. Detecting airborne viruses presents a challenge due to the low concentration of infectious particles, typically ranging from 200 to 600 viral particles per breath [9]. Indoor environments contain approximately 10^14^ particles L^−1^ [10], including both virus-like and bacteria-like particles. Nevertheless, devices enabling real-time assessment of airborne viruses in the environment are now available [11]. These processes involve isolating particles from the air, collecting them in a solution, further concentrating and purifying them, and ultimately identifying breath components using various analytical techniques, such as quantitative real-time polymerase chain reaction (qRT-PCR) for precise and reliable virus detection. However, the integration of bioaerosol samplers with analytical detection techniques for continuous real-time monitoring persists as an ongoing challenge [12].

Nanomaterial-based hybrid nanofibers and sensor arrays are employed to detect and treat VOCs [13–16]. Some of these systems have also proven to be valuable for monitoring specific VOC mixtures from exhaled breath in airborne virus detection [17]. Researchers, such as Cowling et al., have shown the potential for direct airborne virus detection from breath samples [18], often examining viral RNA presence via PCR from condensed exhaled breath [19]. Recently, Ghumra et al. introduced an electrochemical platform for point-of-care testing that directly detects viruses in exhaled breath [9]. Despite considerable advancements, confirming the presence of the virus in exhaled breath without amplification remains uncertain. Limited published data are available on real sample detection of airborne viruses, with biosensing research commonly lacking real-time sensing capabilities due to the typically low pathogen content found in bioaerosols [20].

In this context, the quartz crystal microbalance (QCM) has been employed for the detection of airborne viruses thanks to its excellent sensitivity when studying chemical and biological interfaces in real time [21, 22]. It is one of the choices among many acoustic sensors due to its stability and sensitivity, being a portable low-cost system. For example, the LoDs in a laboratory for this virus in the air were around 40 and 210 pfu/mL at a flow rate of 2.0 and 1.1 L/min, respectively. Moreover, the use of QCM sensors for gas sensing also highlights the interaction between the QCM sensing surface and suspended species in the environmental air [23, 24]. QCM sensors are also widely utilized to selectively detect various targeted gases [25], exhibiting significant potential in advancing sensors for volatile compounds, especially those in low concentrations [26, 27]. The QCM sensor can be modified with different sensitive materials to form a sensor array, optimizing the sensitivity and selectivity of gas detection at room temperature and better realizing the analysis of complex mixtures of gases [28]. Renowned for its cost-effectiveness and firmly established sensing principles, the QCM monitors vibrational changes upon molecular addition to the sensor surface. Through functionalization with antibodies, the QCM chips facilitate the retention and subsequent detection of specific targets, such as proteins or viruses, in solution [29–31]. The biosensor’s selectivity to the chosen target is so conferred by the immobilized antibody.

The fundamental properties of the QCM, such as resonance frequency (*f*) and dissipation factor (*D*), rely on the density and configuration of particles adhered to the sensor surface [32]. As a result, these devices provide real-time responses to the molecular recognition events occurring on the sensor surface, enabling their application in diagnostic tests in solution [21, 29].

In this study, our main objective was to develop a cutting-edge QCM-based breathalyzer, called the “virusmeter.” It has been designed for the explicit purpose of identifying COVID-19 patients by analyzing samples of exhaled air. The device operates without sample treatment or amplification. The research assessed the bioanalytical and medical effectiveness of the virusmeter under controlled conditions, illuminating its potential for enabling self-diagnosis of respiratory diseases, such as COVID-19. Field evaluations were conducted at two public hospitals, a health center, and a nursing home within the Valencia Community, Spain.

## Materials and methods

### Patients and samples

A group of 54 symptomatic COVID-19 patients undergoing hospitalization, alongside 128 healthy participants, provided informed written consent for this study. All volunteers underwent thorough medical examinations and PCR tests upon enrollment. The detection of SARS-CoV-2 genetic markers (N and ORF1ab genes) was performed through RT-qPCR using the Alinity m SARS-CoV-2 assay by Abbott Diagnostics, USA.

Sampling was conducted from February 2nd, 2021, to November 24th, 2021. Regarding the symptomatic COVID-19 patients, 33 were analyzed at the University General Hospital of Castellon, while 21 patients were analyzed at the La Fe University and Polytechnic Hospital in Valencia.

All procedures strictly adhered to established guidelines and regulations. Ethical approvals were granted by the Biomedical Research Ethics Committee of the La Fe University and Polytechnic Hospital in Valencia, Spain, and the University General Hospital of Castellon, Spain. Additionally, the Health Centre “Juana Portaceli” at the Universitat Politècnica de València and the Fortuny nursing home in Valencia, Spain, permitted testing for their interns.

### Chip functionalization

The QCM chips were 5-MHz gold-coated quartz crystal sensors from Renlux Crystal Ltd, China. Before use, the chips underwent the cleaning process detailed in reference [29].

The chips were activated overnight using a 10 mM solution of 3-mercaptopropionic acid (Merck, Darmstadt, Germany). Subsequently, they were immersed in a solution containing 46 mM of N-ethyl-N′-(3-dimethylaminopropyl) carbodiimide (Merck, Darmstadt, Germany) and N-hydroxysuccinimide (Merck, Darmstadt, Germany) for 1 h. Next, the chips were prepared for the covalent immobilization of spike protein (S)-specific antibodies. This process involved dispensing a solution of anti-S antibodies (30 µg·mL^−1^) in phosphate-buffered saline solution (PBS, 8 mM Na_2_HPO_4_, 2 mM KH_2_PO_4_, 137 mM NaCl, 2.7 mM KCl, pH 7.4), prepared using Milli-Q water and filtered through 0.2 polyethersulfone membranes (Merck, Darmstadt, Germany), onto the activated chip surface. The antibodies employed included anti-SARS-CoV-2 spike protein S1 monoclonal antibody HL6 (Genetex, Irvine, USA), SARS-CoV-2 spike protein (S1/S2) recombinant human monoclonal antibody bcb03 (Invitrogen, Waltham, USA), anti-SARS-CoV-2 spike glycoprotein antibody 1A9 (Genetex, Irvine, USA), and anti-SARS-CoV-2 spike protein S1 monoclonal antibody HL1 (Genetex, Irvine, USA). After incubation for 1 h, the chips were rinsed with Milli-Q water and dried in the air stream. Ngo et al. investigated the alterations in the surface morphology due to this treatment [33]. The protein immobilized under these conditions corresponds to a surface concentration of 860 ± 60 ng cm^−2^, as determined in a previous work [34].

### Detection of virus particles in liquid phase

In the optimization of the bioreceptor, changes in frequency (Δ*f*) were monitored using a Q-Sense E1 device (Biolin Scientific, Sweden) that featured a liquid flow cell setup. These experiments were conducted in a solution containing 10^6^ pfu mL^−1^ of SARS-CoV-2 virus-like particles (VLPs) in PBS, at a constant flow rate of 50 µL min^−1^ and a temperature of 25 °C. The Membrane Proteins Lab at the University of Valencia supplied the VLPs utilized in the experiments. VLPs were characterized using a NanoSight Pro, Malvern Panalytical, UK, via NTA measurements interpreted with the NanoSight NTA software v3.3 (see Figure S1 in the Supplementary Material).

### Virusmeter design

**Fig. 1:**
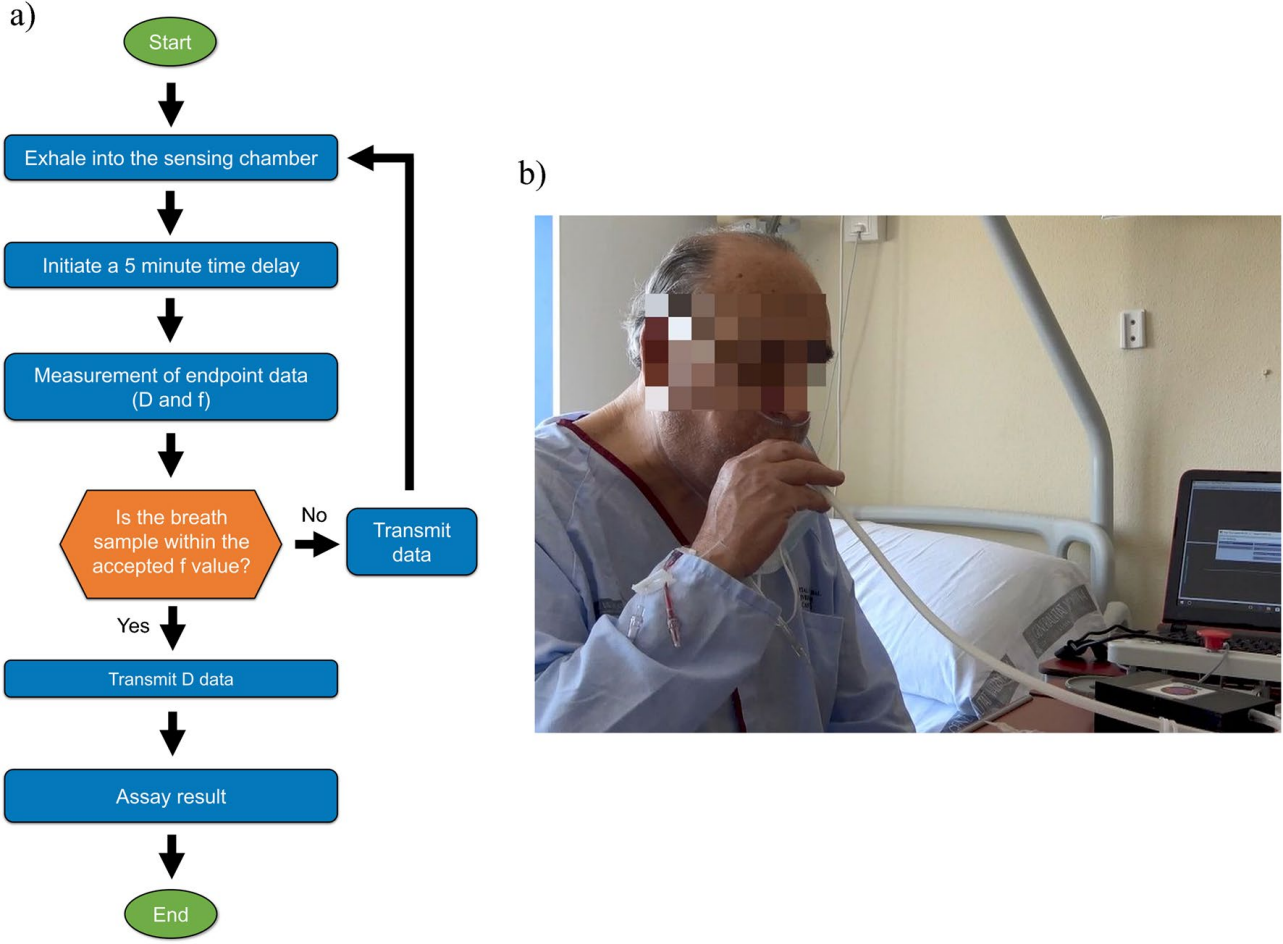
**a** Operational diagram of the virusmeter. **b** Demonstration of breath analysis using the virusmeter on a patient at the University General Hospital of Castellon, Spain

The real-time monitoring of frequency and dissipation values in exhaled air samples was performed using a custom QCM device equipped with an electronic interface based on a customized low-cost vector network analyzer (VNA) [35]. We opted for using this widely accepted impedance-based measurement system due to its established reliability [35–37]. This device exhibits a frequency discrepancy with the commercial OpenQCM Q-1 device of 0.00116 ± 0.00002%, and a dissipation discrepancy of 3.7 ± 0.9%. These measurements were conducted in a controlled environment, with experiments performed over a 60-min interval. Figure 1 shows the operational scheme of the virusmeter: the patient’s exhaled air is directed into the measurement chamber of the transducer box via an anti-return mouthpiece (178NF, C.D.Products S.A., Madrid, Spain) and a 60-cm silicone tube sized 6 × 9 mm (DELTALAB S.L., Barcelona, Spain), as depicted in Fig. 1b. Within the transducer box, viral particles are specifically detected in a label-free manner using an activated QCM chip, allowing real-time monitoring via the VNA. Subsequently, the measurements are recorded on a personal computer (PC).

The vector network analyzer performs passive spectral characterization of the QCM biosensor’s impedance across a spectrum of frequencies around its series resonance [36, 37]. This process enables the identification of the peak conductivity frequency (*f*, series resonance) and the dissipation factor (*D*) of the crystal. The dissipation factor represents the half-power bandwidth of the series resonance peak of the crystal over its resonance frequency.

**Fig. 2:**
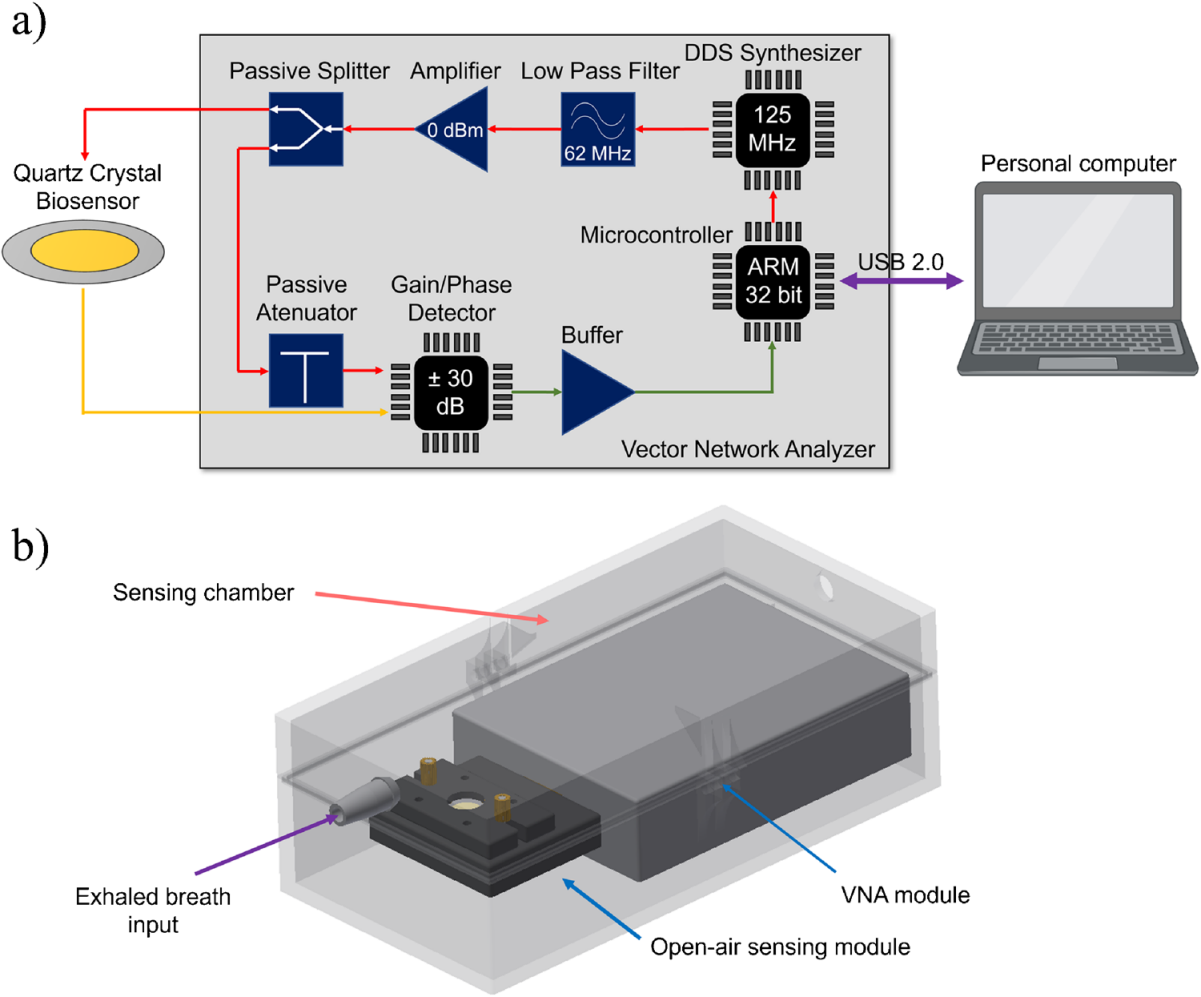
**a** Electronic block diagram of the virusmeter. **b** Schematic representation of the transducer box

Figure 2a shows the electronic block diagram of the device. The direct digital synthesis (DDS) synthesizer, a programmable integrated system, generates the frequency signal for sensor excitation through a high-performance 10-bit digital-to-analog converter, producing analog sinusoidal signals of up to 62.5 MHz. It has a 32-bit frequency tunning register, giving an output resolution of 0.029 Hz. This setup enables sensing of 5-MHz quartz crystals and their 3rd, 5th, 7th, and 9th harmonics. Collaborating with the filter, amplifier, and splitter, the synthesizer delivers a power of 0 dBm to the quartz crystal, maximizing the detector's measurable power.

The detector is an integrated system that measures the gain and phase response between the reference signal (synthesizer signal) and the QCM response. It accommodates an input frequency range spanning from low frequencies up to 2.7 GHz, aligning with the operational frequency of the virusmeter device. Internally, the detector chip features a pair of matched logarithmic amplifiers, offering a measurement gain range of ± 30 dB. Additionally, it hosts a multiplier-type phase detector capable of measuring within the 0 to 180° range, regardless of input signal levels. The external 25-dB attenuator allows for maximizing the dynamic range measurable by the detector between the QCM and reference signals.

The microcontroller, a 32-bit ARM Cortex-M core with added DSP instructions, tunes the frequency sweep in the synthesizer, exciting the QCM biosensor around its resonance frequency. Simultaneously, it digitizes the gain and phase response from the detector for each excitation frequency using the integrated 16-bit analog-to-digital converter modules of the microcontroller. These digitized data are transmitted to a PC via USB 2.0 for real-time analysis, allowing monitoring of characterization parameters like resonance frequency and dissipation factor. Furthermore, the USB port supplies power to the device, with a maximum current of 250 mA and a voltage of 5 Vdc, making it a Plug&Play capable device.

Figure 2b illustrates the components within the transducer box, crafted by means of a 3D printer (Ultimaker 2 Extended, Ultimaker B.V, Utrecht, Netherlands) using polylactic acid (PLA) material (125–4336, RS Pro, London, UK). The box measures 77 mm × 152 mm × 50 mm (width × length × height) with an internal chamber volume of 168.2 cm^3^. Comprising two sections depicted in Fig. 2b, the bottom segment houses the VNA module and the free-air module of the QCM biosensor. Meanwhile, the top sensing chamber functions as the inlet for exhaled air. These two parts are secured by a U-shaped slot along the perimeter for sealing and held together by pins on their sides.

A custom PC-based software oversees real-time monitoring of QCM parameters, delivering analytical insights to users. This software establishes communication with the VNA through the USB interface, configures the sweep frequencies for odd harmonics sensed in the VNA, and collects the resultant data. Upon collection, the software generates the gain and phase curves of the sensor frequency response, smoothed using the Savitzky-Golay filtering algorithm [38, 39], effectively reducing noise while preserving signal integrity. It then identifies the peak conductance, extracting the associated resonance frequency and dissipation values in real time. These values correlate with changes in data evolution (*f* and *D*), indicative of adsorbed species on the QCM sensor. The graphical user interface displays this data and maintains a log for measurement history.

### Use of the virusmeter

After functionalization, the sensing chip was positioned with the coated side facing up in the open-air module of the QCM device. The system underwent calibration and stabilization until the 3rd harmonic frequency and dissipation factor reached steady baselines. Subsequently, while seated, each participant exhaled into the sensing chamber (refer to Fig. 1). To ensure proper air sampling, a protocol was established where individuals were instructed to exhale with an expiratory flow of 320 L min^−1^ into the disposable sampling tube twice for 3–5 s while maintaining a consistent stream. This instruction was necessary due to the significantly higher flow rate than the typical exhaled breath volume for adults, around 6 L min^−1^ [40]. This flow resulted in an exhaled air volume of 16,000–27,000 cm^3^ into a chamber volume of 168.2 cm^3^, reducing protein loss in the environment due to non-specific adsorption on the virusmeter walls. The first blow cleared residual air from the chamber, and the subsequent one ensured that air from the lower respiratory tract was collected, going beyond the usual collection from the upper respiratory tract in classical assays.

Participants were also advised not to eat, drink, or smoke for at least 40 min before the test. Fasting beforehand was also discouraged as it could influence the composition of exhaled air. These controlled conditions are vital, given that volatile organic compounds may non-specifically adhere to the functionalized chips, potentially affecting the measurement signal [21]. Real-time acquisition of frequency and dissipation factor data took place over the following 5 min. Finally, endpoint data were compared to the baseline to determine the net frequency and dissipation factor shifts (denoted as Δ*f* and Δ*D*, respectively) within a 5-min interval following exhalation. A new chip was used for each measurement.

It is crucial to reiterate that, before each exhalation, the baseline signals of the virusmeter were stabilized and remained constant throughout the brief 5-min measurement period. In longer-duration applications of this sensor, similar to many analytical methods, the signal may experience instrumental drift, but this drift can be successfully eliminated using compensation algorithms during post-processing of the data [41].

## Results and discussion

### Optimization of the bioreceptor in liquid phase

During the timeframe of this study, the prevailing variants of SARS-CoV-2 in Spain were Alpha, Delta, and Omicron. In order to select the optimal capture antibody for the virus, four antibodies (HL6, bcb03, HL1, and 1A9) targeting the SARS-CoV-2 spike glycoprotein underwent further characterization through direct immunoassay. These antibodies, immobilized on QCM’s chips, were exposed to a 10^6^ pfu/mL solution of SARS-CoV-2 virus-like particles (VLPs) for 1 h to stabilize the signal and ensure the measurement of the maximum surface concentration of VLPs under these experimental conditions. The signal shift was monitored from the onset, and the test concentration chosen in this study corresponds to the typical limit of detection (LoD) achieved by biosensors designed for bioaerosol analysis [42]. Subsequently, the interaction was monitored using quartz crystal microbalance with dissipation (QCM-D), revealing a consistent decrease in frequency across all assays.

The acoustic response of the QCM-D was assumed to mimic that of an ultrathin layer in a Newtonian bulk liquid [32, 43]. Hence, the observed decrease in Δ*f* suggests a rise in the surface concentration of the S protein-antibody complex at the fluid-solid interface.

The variability among antibodies in their response to VLPs was evident in Δ*f*, closely linked to protein affinity. In the characterization study, the monoclonal anti-S antibody HL6 displayed Δ*f* =  − 6.8 Hz, while bcb03 showed − 3.2 Hz, HL1 − 1.2 Hz, and 1A9 − 0.8 Hz, with an error margin of ± 0.5 Hz under these experimental conditions, maintaining a baseline signal of 0.4 ± 0.5 Hz. These data confirm the formation of the S protein-antibody complex on the sensor surface, highlighting HL6’s superior affinity for the SARS-CoV-2 spike glycoprotein present in our VLPs and justifying its selection for immobilization on the sensor surface in the developed detection system. According to the commercial provider, this antibody has a limit of detection for the recombinant SARS-CoV-2 (COVID-19) spike S1 subunit protein of 62.5 ng mL^−1^ and shows no cross-reactivity with SARS-CoV or MERS-CoV spike proteins [44]. However, it is crucial to note that mutations within the targeted glycoprotein across various virus variants can induce fluctuations in protein affinities, resulting in distinct signals even when monitoring the same viral concentrations. Therefore, controlling the study conditions is paramount for this proof-of-concept.

### Experimental performance of the virusmeter

Initiating the practical application phase, we will monitor the complex composition of exhaled air, encompassing up to 200 compounds, including endogenous factors derived from the host’s metabolism and exogenous elements like virions or microbiota [45]. Consequently, in the collected exhaled air samples, our transducer’s signal cannot be exclusively attributed to the interaction between the SARS-CoV-2 virus and specific antibodies immobilized on the sensor surface. It also reflects the interaction of all exhaled compounds with the sensor. These secondary signals are frequently observed in clinical devices, such as those employed for the fractional exhaled nitric oxide (FeNO) test, which measures nitric oxide levels in exhaled breath to assess bronchial inflammation in asthma patients. Therefore, each personal exhalation is unique and influenced by multiple factors.

The transducer and exhaled air interplay will induce shifts in the crystal’s fundamental resonant frequency (Δ*f*) and viscoelastic properties (represented as dissipation factor, Δ*D*), directly correlated with the patient’s diagnosis. Real-time monitoring of both parameters will facilitate highly sensitive and quantitative measurement of biorecognition events. Interactions involving large molecules such as virions lead to notable viscoelastic changes [43], significantly facilitating the detection of samples with exceptionally low target concentrations, as evidenced in our case.

Based on insights from Lee et al. [21], it is reasonable to propose that the sensing process starts with the adsorption of bioaerosols containing viral particles onto the functionalized sensor surface, promoting the interaction between the bioaerosol and the immobilized antibody. Subsequently, the adsorbed aerosol evaporates rapidly due to its high internal pressure, remaining the biomolecules which interact with the immobilized bioreceptor onto the chip surface, leading to changes in Δ*D* values.

**Fig. 3:**
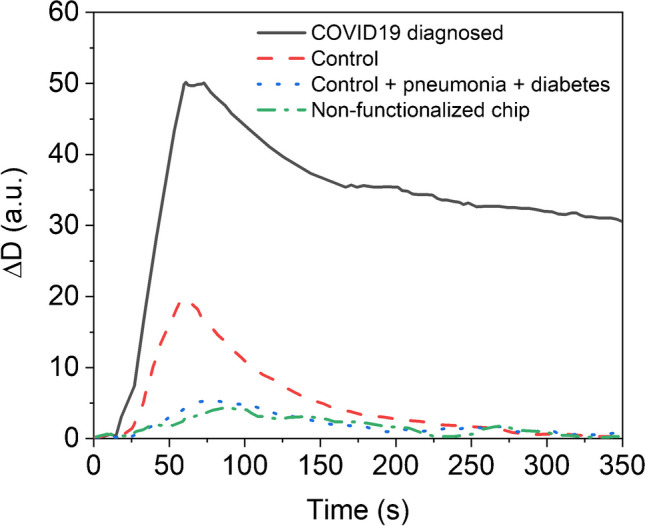
Real-time dissipation factor signals obtained after analyzing a COVID-19 hospitalized symptomatic patient diagnosed via nasopharyngeal PCR with 30 thermal cycles (solid black line), a healthy control volunteer (dashed red line), a patient with diabetes and pneumonia unrelated to SARS-CoV-2 (dotted blue line), and the exhaled air analysis of a COVID-19 diagnosed patient using a not functionalized sensor chip (dashed green line)

To evaluate the sensor’s ability to distinguish between symptomatic patients and healthy individuals, both groups underwent testing with the virusmeter. In Fig. 3, it is evident that, in all cases, the Δ*D* signal increases and stabilizes within 5 min after exhalation, showing a minimal change of only 10% after 60 min. Consequently, the virusmeter’s endpoint Δ*D* signal was defined as the signal measured 5 min after exhalation. The difference in stabilization time between the experimental liquid (60 min) and gas phase (5 min) arises because the exhaled air dissipates from the sensing chamber after 5 min due to indoor air regeneration, as the chamber is not sealed. In these experimental conditions, the endpoint Δ*D* observed in a symptomatic patient (Δ*D* ≈ 30.3 a.u., solid black line) was notably higher—up to 60 times—than the signal from healthy control subjects (Δ*D* ≈ 0.5 a.u., dashed red line). Furthermore, and to assess the biosensor’s selectivity, we examined the breath sample of a patient with diabetes and pneumonia unrelated to SARS-CoV-2 (blue line in Fig. 3), resulting in an endpoint Δ*D* of 0.0 a.u. This result highlights the virusmeter’s efficacy in utilizing the endpoint Δ*D* signal to discriminate between symptomatic patients and control subjects.

In terms of Δ*f*, the signal decreases to a minimum and returns to the initial value within 5 min. The endpoints observed for patients (Δ*f* ≈ 0.5 Hz) remained below the system’s quantification limit (± 1 Hz). Nevertheless, the value of the observed minimum is strongly influenced by the quantity of exhaled air, as well as the Δ*D* value. For instance, if the minimum Δ*f* does not exceed − 2 Hz, the endpoint Δ*D* reads 0.8 a.u. in a symptomatic patient. However, it increases to 6.0 a.u. when Δ*f* falls between − 2 and − 5 Hz. Although Δ*f* might not be as sensitive as Δ*D* in detecting airborne viruses, real-time monitoring is crucial during exhalation. Precise sampling requires Δ*f* to ideally reach a minimum value between − 2 and − 5 Hz, since a blow with values below − 5 Hz causes sensor calibration issues, whereas values above − 2 Hz indicate very few particles reaching the sensor. This final conclusion is a typical behavior observed in comparable systems like breathalyzers. If an inadequate amount of exhaled air is introduced into the sensor, the sensor will fail to detect any substance. Thus, real-time monitoring of Δ*f* ensures the evaluation/validation of the exhalation process, providing control over assay conditions (as commented above, an expiratory flow around 320 L min^−1^. This value refers to the peak expiratory flow (PEF) recorded with a spirometer for an 80-year-old woman who is 160 cm tall), achieving a reproducibility between consecutive blows of 7%. Different exhalation techniques were also examined, normal and diaphragmatic, to evaluate their effects on the virusmeter’s endpoint Δ*f* signal. When a control subject exhales normally, the virusmeter signal measures 3 ± 0.5 Hz. However, during diaphragmatic breathing, Δ*f* increases more than 16 Hz, causing the sensor to cease resonating correctly. Higher exhalation rates lead to a decrease in the QCM sensor’s resonance frequency [46]. This dual measurement approach, where expiratory flow is controlled simultaneously with the measurement, mirrors the methodology often seen in clinical devices like those utilized for the FeNO test.

The impact of attached anti-S antibodies on the virusmeter signal was also investigated. In Fig. 3, the exhaled air analysis of a symptomatic patient is depicted when the sensing chip is not functionalized (represented by the green dashed line). Under these conditions, the endpoint Δ*D* observed for symptomatic patients was comparable to controls (Δ*D* ≈ 0.8 a.u.). Consequently, patients and control subjects are not distinguishable when the sensing chip is not functionalized. These findings emphasize that the signals observed when chips are functionalized are inherently associated with the utilized bioreceptor. These signals do not stem from changes in the temperature or humidity of the exhaled air, given that the variation in temperature and humidity between the beginning and the end of the experiment is zero. Therefore, neither parameter affects the endpoint data.

**Fig. 4:**
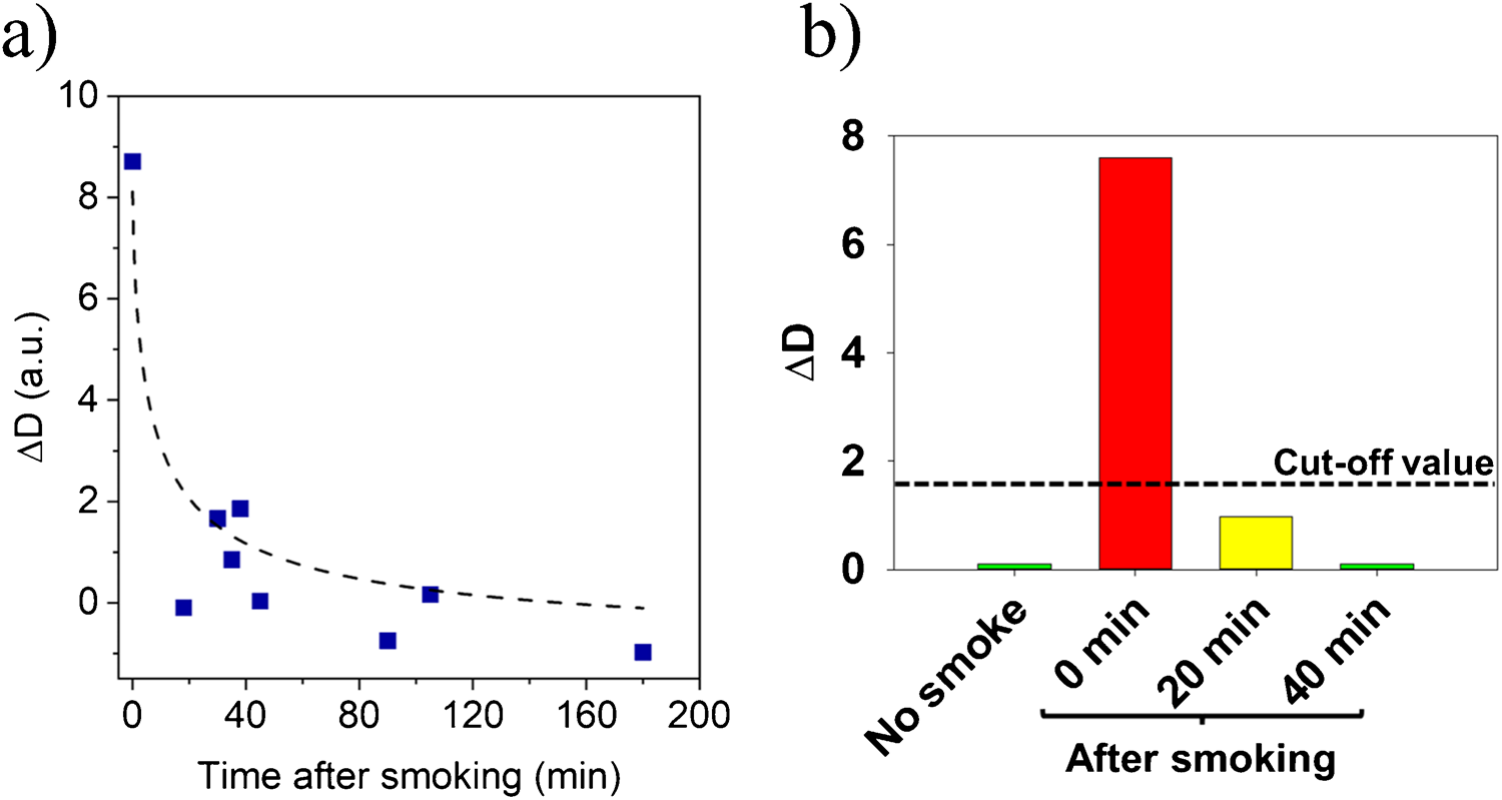
**a** Virusmeter response of 12 heavy smokers grouped at different post-smoking time intervals. **b** Virusmeter timeline depicting a user’s breath analysis post-smoking

It is well established that the exhaled air of smokers differs significantly from that of non-smokers, primarily due to the exhalation of specific organic volatile compounds (VOCs) [47]. Tobacco smoking introduces hundreds of harmful substances into the lungs through voluntary inhalation, establishing a dose–response relationship between smoking intensity and VOC metabolites in exhaled breath [47]. These compounds could potentially interfere with the accuracy of virusmeter measurements, as commented above. To address this concern, we selected 12 heavy smokers volunteers who tested negative for COVID-19/SARS-CoV-2 via PCR and were clinically confirmed as healthy. In the case of smokers, samples of exhaled air were analyzed using the virusmeter at various time intervals before and after smoking, as illustrated in Fig. 4a. For example, Fig. 4b depicts the temporal progression of the endpoint Δ*D* signal recorded after assessing a smoker control. In all 12 cases, the Δ*D* signal increases to approximately 8 a.u. immediately after smoking and then gradually decreases in varying patterns until reaching the pre-smoking value after 40 min, stabilizing around 0.0 a.u. Hence, to ensure the reliability of exhaled air measurements, it is crucial to implement a waiting period of approximately 40 min after smoking before conducting a virusmeter test, assuming a cutoff of Δ*D* ≅ 1.55 a.u.

Similarly, consuming liquids before the test may introduce uncertainty, as they can clear the upper respiratory tract. For instance, an endpoint Δ*D* signal of around 28.40 a.u. dropped to 0.08 a.u. immediately after the user drank water, rebounding to 25.30 a.u. after 10 min. This experimental result highlights the intricacy of exhaled air samples and demonstrates that controlling the conditions under which the test is performed is crucial. Hence, individuals undergoing testing were advised to refrain from eating, drinking, or smoking for about 40 min, aligning with commercial self-test guidelines [48].

With the aim of studying the reusability of the sensing chips, several measurements were done, in similar conditions for specific individuals, under their consent. For control subjects, chips remained fully functional after 26 measurements, with an average Δ*D* of 0.2 ± 0.4 a.u. Deviations exceeding 1 a.u. were observed beyond this threshold (see Figure S4). However, for patients, the endpoint Δ*D* significantly decreases after the first exhalation (see Figure S4). This last variation cannot be attributed to viral load since it pertains to the same patient and returns to its initial value when the chip is replaced. For that reason, it was decided to employ a new chip for each measurement.

As shown in Table 1, most hospitalized patients experienced respiratory distress, posing challenges in obtaining consent for rigorous, multi-measurement procedures for each individual. Additionally, as outlined before, individual exhalations are influenced by various factors, making each one distinct for the same individual. However, the reproducibility of virusmeter signals was also evaluated for specific symptomatic patients who provided consent. Thus, four symptomatic diagnosed patients underwent testing in triplicate using three different sensor chips, obtaining 10.3 ± 1.3, 20.5 ± 1.1, 2.3 ± 0.3, and 2.78 ± 0.02 a.u. in Δ*D*. The RSD of these assays varied from 0.7 to 13.0%.

**Table 1:** Characteristics of the study population: Note that patients are often poly-symptomatic

	Clinical diagnosis	
	Positive	Negative
No. (% of total)	54 (30)	128 (70)
Demographic characteristics		
Age, average ± SD (range)	40 ± 16 (23–88)	23 ± 25 (16–97)
Female sex, No. (% of grouping)	25 (46)	73 (57)
Male sex, No. (% of grouping)	29 (54)	55 (43)
Total, No. (% of grouping)	54 (100)	128 (100)
Age by groups		
Young adults (16–44), No. (% of grouping)	24 (45)	83 (65)
Old adults (45–64), No. (% of grouping)	21 (38)	19 (15)
Seniors (65 or older), No. (% of grouping)	9 (17)	26 (20)
Total, No. (% of grouping)	54 (100)	128 (100)
COVID-19 vaccination		
COVID-19 vaccinated, No. (% of grouping)	3 (6)	26 (20)
COVID-19 not vaccinated, No. (% of grouping)	51 (94)	102 (80)
Total, No. (% of grouping)	54 (100)	128 (100)
Clinical status of COVID-19 patients		
* Mild*		
No. (% of grouping)	16 (30)	
Age, average ± SD (range)	38 ± 16 (23–76)	
* Moderate*		
No. (% of grouping)	30 (55)	
Age, average ± SD (range)	61 ± 16 (28–88)	
* Severe*		
No. (% of grouping)	8 (15)	
Age, average ± SD (range)	52 ± 13 (30–71)	
Total, No. (% of grouping)	54 (100)	
Clinical symptoms: patients with multiple symptoms		
Dyspnea, No. (% of grouping)	21 (39)	
Smell dysfunction, No. (% of grouping)	13 (24)	
Taste dysfunction, No. (% of grouping)	9 (17)	
Fever, No. (% of grouping)	7 (13)	
Muscle pain, No. (% of grouping)	3 (6)	
Cough, No. (% of grouping)	2 (4)	
Headache, No. (% of grouping)	12 (22)	
Asthenia, No. (% of grouping)	2 (4)	
Pneumonia, No. (% of grouping)		4 (3)
Diabetes, No. (% of grouping)		5 (4)

Finally, concerning the operation conditions of the system, it is well known that temperature and humidity influence the QCM sensor. Nevertheless, it is important to note that the final measurement occurs once the system stabilizes, typically within 5 min. During this stabilization period, the initial conditions revert to their original state due to indoor air regeneration (i.e., Δ*T* and ΔRH = 0). Thus, fluctuations in these variables during the sampling stage of the virusmeter do not affect the final measurement outcome. Consequently, to simplify interpretation of our sensor, additional parameters were not introduced. In previously reported studies on detecting airborne viruses in exhaled breath using conductimetric methods [17, 21], for similar reasons, these parameters were not monitored on a QCM-based sensor for Vaccinia virus in air.

### Clinical evaluation

Following the demonstration of the capability of the virusmeter in identifying COVID-19 patients by exhaled air analysis, a comprehensive clinical evaluation was conducted. A total of 54 symptomatic patients with confirmed clinical diagnoses and 128 negative controls were included in the analysis. The average age of the patients was 40 years (ranging from 23 to 88 years old), being 46% female and 54% male. Among the symptomatic participants, 30% had mild illness, 56% had moderate, and 14% experienced severe illness. Common symptoms included dyspnea (39%), smell dysfunction (24%), taste dysfunction (17%), fever (13%), muscle pain (6%), cough (4%), headache (22%), and asthenia (4%). A significant proportion of patients (86%) presented a high viral load (PCR Ct < 30, N gene). The patient selection aimed to guarantee that positive exhaled air samples had virions, based on Johnson et al.’s discovery not all with positive nasopharyngeal RT-qPCR results have infectious viruses in their exhaled breath [49]. The control group comprised individuals vaccinated against COVID-19 (21%), individuals with pneumonia associated with other diseases (4%), individuals with diabetes (5%), and healthy individuals (70%). A summary of the characteristics of both patients and controls is provided in Table 1.

**Fig. 5:**
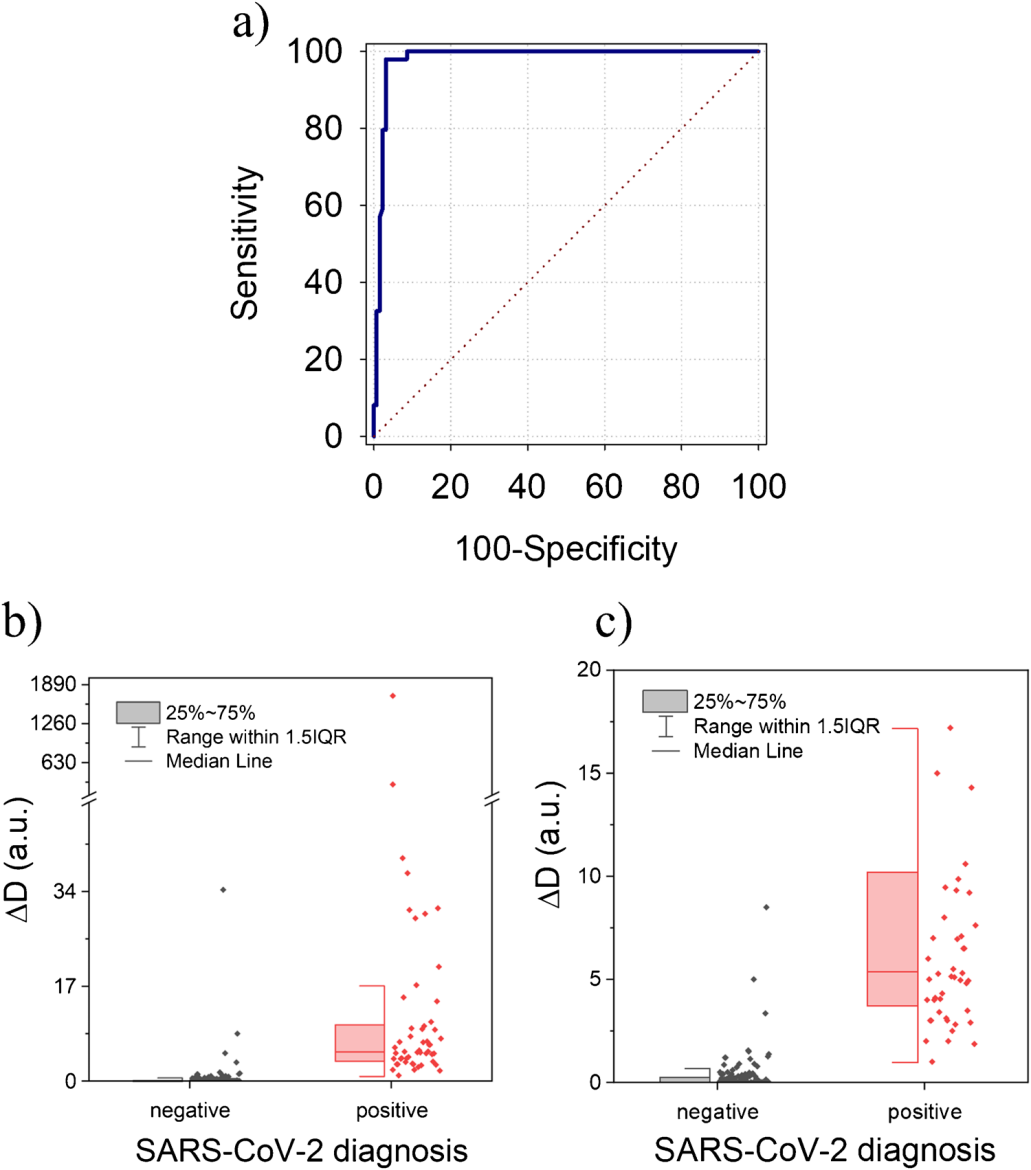
**a** ROC curve for prediction of COVID-19 based on the virusmeter response. **b** The grouped half-box indexed plot shows the virusmeter levels in the symptomatic patients diagnosed positive and negative. **c** Zoomed view of the results presented in (**b**), ranging from 0 to 20 a.u.

In Fig. 5a, a receiver operating characteristic (ROC) curve is constructed using our experimental data, comparing clinical diagnoses, conducted by physicians and confirmed by nasopharyngeal RT-qPCR tests (gold standard), with virusmeter data to establish the true-positive rate in relation to the false positive rate. The area under this ROC curve (AUROC) serves as a measure of accuracy, indicating the system’s ability to differentiate between groups. For the virusmeter, AUROC was 0.982 (95% CI 0.950 − 0.996), suggesting that the developed system possesses excellent predictive capacity in subjects with a high viral load. Additionally, the analysis of covariance (ANCOVA) yielded *p* values below 0.0001, signifying a robust statistical significance in the difference between the virusmeter results of symptomatic patients with confirmed diagnosis and control subjects, as depicted in Fig. 5b and c where the statistical dispersion of both groups is illustrated using the interquartile range. A real-time acquisition of raw frequency and dissipation data for a representative number of patients and controls (np = 25; nc = 25) can be analyzed in Figures S2 and S3 in the Supplementary Material.

Moreover, it is important to note that the IQR method plays a crucial role in identifying outliers statistically. It establishes a “fence” beyond Q1 and Q3, with values outside of this fence considered outliers. The fence is constructed by multiplying the IQR by 1.5 and subtracting this value from Q1 while adding it to Q3. These minimum and maximum fence posts are then used to compare each observation. Any observation lying more than 1.5 times the IQR below Q1 or above Q3 is classified as an outlier. This method is illustrated in Fig. 5b and c to emphasize the statistical distinction between both groups. Furthermore, a box illustrates the range from the 25th to the 75th percentiles for both control subjects and patients in these figures. Based on these boxes, we can assert that our equipment’s measurement capability facilitates a clear differentiation between these groups.

Evaluation metrics in Table 2, based on the ROC curve, highlight the efficacy of our binary classifier system to identify COVID-19 patients. The optimal cutoff for the virusmeter, associated with the Youden’s index (maximum sum of sensitivity and specificity), was 1.55 a.u., achieving a sensitivity of 98.15% (95% CI 90.1–100.0) and a specificity of 96.87% (95% CI 92.2–99.1).

**Table 2:** Performance metrics of different virusmeter testing for predicting the given record as positive when tested on external validation

Performance measure	Confirmed diagnosis vs virusmeter
AUC	0.982
Accuracy	0.968
F1 score	0.947
Sensitivity (recall)	0.981
Specificity	0.969
Positive predictive value (precision)	0.982
Net present value	0.962
False positive rate	0.008
False discovery rate	0.018
False negative rate	0.085

Although the RT-PCR assay for measuring SARS-CoV-2 is designed to detect viral RNA, offering a positive result that indicates the presence of viral nucleic acid, it cannot detect viruses in the lower respiratory tract without lung sampling and cannot differentiate between viable and nonviable viruses. In line with this, there is no clear correlation between virusmeter measurements and the thermal cycles of nasopharyngeal PCR measurements alone (measurements in the upper respiratory tract). This fact is aligned with CDC guidance that Ct values do not reflect infectiousness across SARS-CoV-2 variants [48].

Quantitatively, virusmeter measurements seem linked to an individual’s infectivity level and disease severity. Given the sensor’s design, which is functionalized with specific bioreceptors, it is reasonable to assume that its results are more closely linked to the virion quantity in the exhaled air sample than the patient’s Ct values. However, further studies are required to delve into this association.

The target population for utilizing this testing method was subsequently defined, taking into consideration the clinical status of the patients. Symptomatic patients with confirmed COVID-19 diagnoses were categorized into three illness levels: mild, moderate, and severe. Mild illness encompassed individuals exhibiting symptoms without dyspnea. Moderate illness included those showing evidence of lower respiratory disease with oxygen saturation of equal or above 94%, while severe illness comprised individuals with oxygen saturation below 94%. The virusmeter exhibited the highest sensitivity in identifying patients with moderate and severe illness, achieving a sensitivity of 100%. For mild symptoms, the sensitivity was 93.75%, which aligns with expectations, given the potential association of higher viral loads with severe clinical outcomes [50]. Consequently, the virusmeter accurately identifies patients with moderate and severe illness, although its utility with mild patients with high viral load should not be overlooked. Importantly, the specifications of the virusmeter meet the criteria set by the World Health Organization, with sensitivity exceeding 80% and specificity exceeding 97% compared to nucleic acid detection tests [51]. Notably, data suggest that the virusmeter would demonstrate high potential in identifying infected with a high viral load.

Compared to RT-PCR nasopharyngeal tests, among the 128 individuals diagnosed as negative, 123 tested negative for the virusmeter, with only 5 showing positive results, resulting in an overall negative predictive value of 96.09% (95% CI 95.62–99.98). Conversely, among the 54 patients diagnosed as positive, 53 had concordant results between the nasopharyngeal diagnosis and the virusmeter test, with only one negative result, yielding an overall positive predictive value of 98.20%. It is crucial to highlight that three individuals who tested negative by nasopharyngeal RT-PCR were positive by the virusmeter and were subsequently diagnosed as positive through more invasive techniques, such as RT-PCR tests from sputum or bronchial aspirate samples. This fact underscores the importance of the reference sampling technique in validating the virusmeter, as these cases were detected through a comprehensive study of the entire respiratory tract.

### Special cases

As is common in almost all diseases, the clinical profile of the patients was highly heterogeneous. Among control subjects, a notable observation was the signal provided by a 21-year-old man who initially tested positive with the virusmeter testing, showing an endpoint Δ*D* = 30 a.u. However, the next day, he tested negative by both the virusmeter and nasopharyngeal RT-PCR, yet still tested positive for the COVID-19 virus serological test. Physicians interpreted these results as his body actively fighting off the infection or having already overcome it.

The first notable case involved a patient with a history of HIV since 1999, admitted to the hospital with symptoms such as fever (38 °C), general malaise, cephalgia, irritative cough, and diarrhea, all within the context of a SARS-CoV-2 infection diagnosed by nasopharyngeal PCR. This patient turned out to be a superspreader, as her family and cohabitant partners were infected, displaying similar symptoms within a few days.

**Fig. 6:**
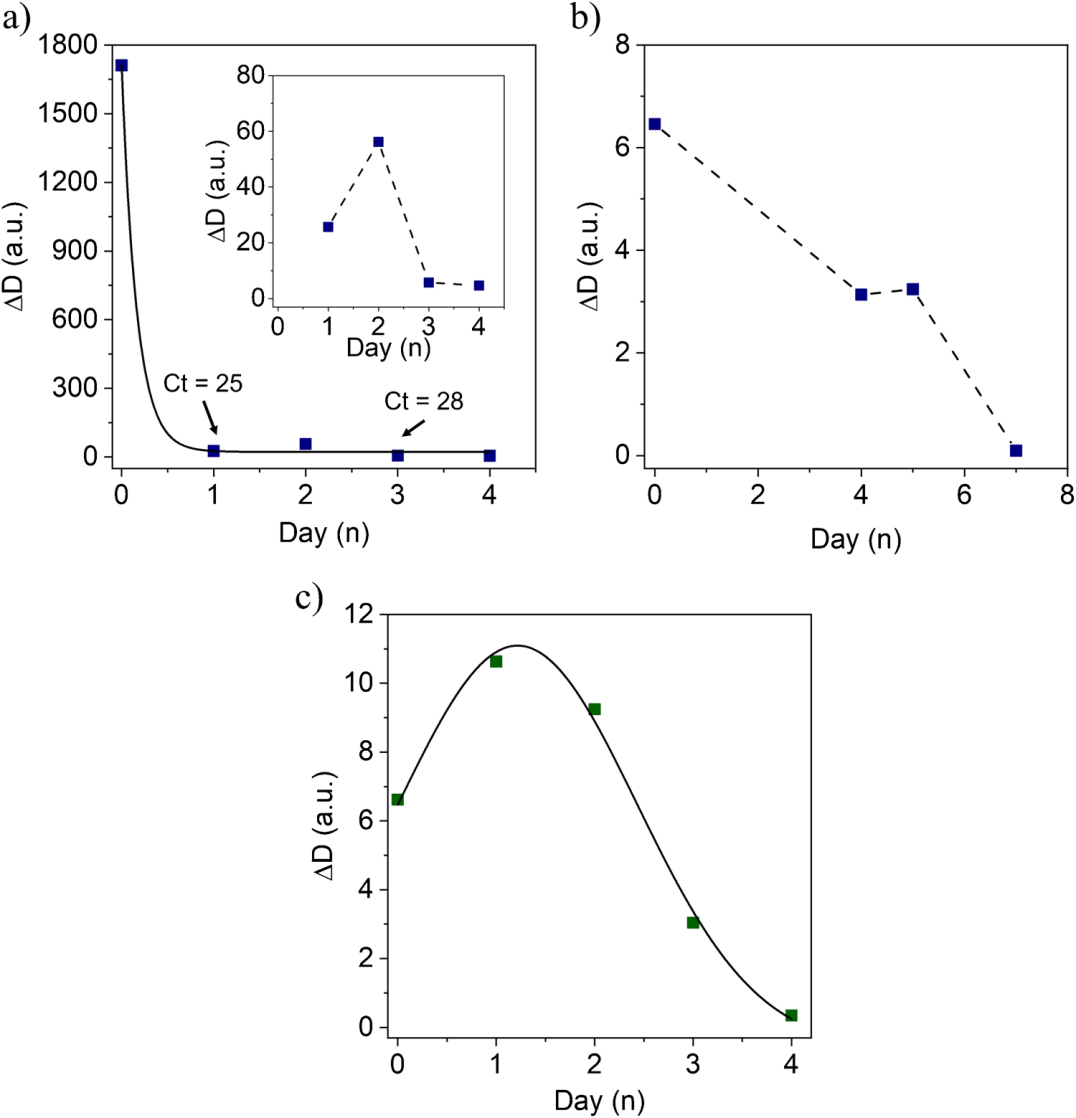
**a** Follow-up study on a hospitalized superspreader COVID-19 positive-diagnosed patient. The inset in this figure provides a close-up view of the virusmeter results from the second day of hospitalization. **b** Disease regression monitoring during the hospitalization of a diagnosed COVID-19 patient. **c** Self-sampling of disease regression in a non-trained individual clinically diagnosed as COVID-19 positive

Upon admission, she underwent her first virusmeter test, registering a high Δ*D* signal of 1700 a.u., as shown in Fig. 6a. The following day, after stabilizing clinically with her regular retroviral medication, she performed a second virusmeter test, yielding a smaller signal of the endpoint Δ*D*, around 30 a.u. This reduction suggested that medication and stabilization would significantly decrease the concentration of virions. A gradual diminution of signals was observed in the subsequent days until reaching a signal below the cutoff value on the fourth day.

In specific cases where initial testing of upper respiratory tract samples yields negative results despite clinical symptoms suggestive of a SARS-CoV-2 infection, conducting an RT-PCR with deep sampling may be necessary for an accurate diagnosis. This fact is because a higher viral load tends to be retained in the lower respiratory tract [52, 53]. In our study, we encountered two noteworthy cases that underscored this phenomenon. The first case involved a 70-year-old man admitted to the hospital with fever, non-productive cough, and dyspnea. He initially tested positive for COVID-19 via RT-qPCR. Days later, the virusmeter test was positive, but a follow-up nasopharyngeal PCR on the same day came back negative. Nevertheless, as the patient’s symptoms progressed, necessitating a transfer to the intensive-care unit, he eventually tested positive in a bronchoalveolar lavage, confirming that the viral load was predominantly concentrated in his lungs. This outcome confirms the validity of the signal acquired through the entire respiratory tract sampling conducted with the virusmeter test.

Similarly, a second patient, a pregnant woman experiencing dyspnea and a dry cough, underwent a nasopharyngeal RT-PCR that yielded a negative result, despite her clinical presentation aligning with COVID-19. The virusmeter test, however, indicated a positive result. Consequently, healthcare professionals continued to investigate the case, and after 2 days, a COVID-19 diagnosis was confirmed through an RT-PCR test using sputum specimens. A bronchial aspirate sampling was not conducted due to its invasive nature and the associated risk of preterm delivery.

Due to its user-friendly design and accurate testing capabilities, the virusmeter can serve as a continuous monitoring tool for hospitalized patients, as illustrated in Fig. 6b. Additionally, the virusmeter allows for daily self-monitoring of disease regression by non-trained individuals with a high viral load, as depicted in Fig. 6c. This self-testing approach not only reduces the risk of infection for healthcare workers but also opens up a range of clinical applications, including assessments of infectivity and determination of the infection phase.

## Conclusions

In summary, we developed a QCM-based breathalyzer, named the “virusmeter,” specifically engineered to differentiate between individuals with respiratory diseases exhibiting symptoms and healthy individuals. This process is completed within a swift 5-min timeframe, eliminating the need for additional sampling steps. The sensor captures exhaled air from the entire respiratory tract. To address the complexity of exhaled air samples, the proof-of-concept was conducted under meticulously controlled experimental conditions. Participants were instructed to refrain from eating, drinking, or smoking for at least 40 min before the test to prevent potential interference of VOCs with the sensor’s measurements.

The diagnostic capacity of the developed equipment was then assessed in a restricted population, comprising 54 symptomatic COVID-19 patients undergoing hospitalization with high viral loads, as well as 128 negative controls. Under these controlled conditions, the sensor demonstrated good clinical specificity (96.87%, 95% CI 92.2–99.1) and sensitivity (98.15%, 95% CI 90.1–100.0) for exhaled air-based diagnosis in the tested population (*N* = 182). Concerning the potential use for quantifying the viral load, virusmeter values appear to be associated with individual infectivity and disease severity, necessitating additional investigation.

This proof-of-concept introduces a promising method for utilizing piezoelectric sensors to diagnose respiratory diseases. The virusmeter, characterized by its affordability, sensitivity, selectivity, user-friendliness, and rapidity, provides an accessible method for end-users. Moreover, the equipment’s adaptability enables the modification of the bioreceptor to detect different variants of the same virus or even different viruses, which opens new avenues toward its application in several sanitary contexts.

## Supplementary Information

Below is the link to the electronic supplementary material.

Supplementary file1 (DOCX 762 KB)

## Declarations

### Source of biological material

Exhaled air samples were monitored on-site at several locations, including La Fe University and Polytechnic Hospital in Valencia, University General Hospital of Castellon, Health Centre at the Universitat Politècnica de València, and Fortuny nursing home in Valencia. Sampling occurred from February 2nd, 2021, to November 24th, 2021. A total of 33 patients were analyzed at the University General Hospital of Castellon, and 21 patients were analyzed at La Fe University and Polytechnic Hospital in Valencia, totaling 54 patients. Control subjects included 84 individuals from the UPV Health Centre “Juana Portaceli,” 25 from the Fortuny nursing home, 12 from La Fe Hospital, and 7 from the Hospital of Castellon, totaling 128. Special measures were taken to prevent the spread of the virus during sampling.
