# Supplementary material for: Real-world evaluation of a QCM-based biosensor for exhaled air
Source: Anal Bioanal Chem. 2024 Jun 26;416(30):7369–83. doi: 10.1007/s00216-024-05407-5 (PMC11584482; doi:10.1007/s00216-024-05407-5)
Supplement: Supplementary file 1 — Supplementary file1 (DOCX 762 KB) [file 216_2024_5407_MOESM1_ESM.docx]

**Supplementary Material**

**Real-world Evaluation of a QCM-based Biosensor for Exhaled Air**

Augusto Juste-Dolz^a^, William Teixeira^a^, Yeray Pallás-Tamarit^a^, Mario Carballido-Fernández^b,c^, Javier Carrascosa^a^, Ángela Morán-Porcar^b^, María Ángeles Redón-Badenas^b^, María Gracia Pla-Roses^b^, María Dolores Tirado-Balaguer^b^, María José Remolar-Quintana^b^, Jon Ortiz-Carrera^d^, Ethel Ibañez-Echevarría^d^, Angel Maquieira^a,e^*, David Giménez-Romero^f^*

^a^ Instituto Interuniversitario de Investigación de Reconocimiento Molecular y Desarrollo Tecnológico (IDM), Universitat Politècnica de València, Universitat de València, Camino de Vera s/n, 46022 Valencia, Spain.

^b^ Hospital General Universitario de Castellón, Avinguda de Benicàssim, 128, 12004, Castellón de la Plana, Spain.

^c^ Universidad CEU Cardenal Herrera, Calle Grecia, 31, 12006, Castellón de la Plana, Spain.

^d^ La Fe University and Polytechnic Hospital, Avinguda de Fernando Abril Martorell, nº 106, 46026, Valencia, Spain.

^e^ Departamento de Química, Universitat Politècnica de València, Camino de Vera s/n 46022, Valencia, Spain.

^f^ Departamento de Química-Física, Universitat de València, Calle Doctor Moliner 50, 46100, Burjassot, Spain.

* authors for correspondence

(a)

(b)


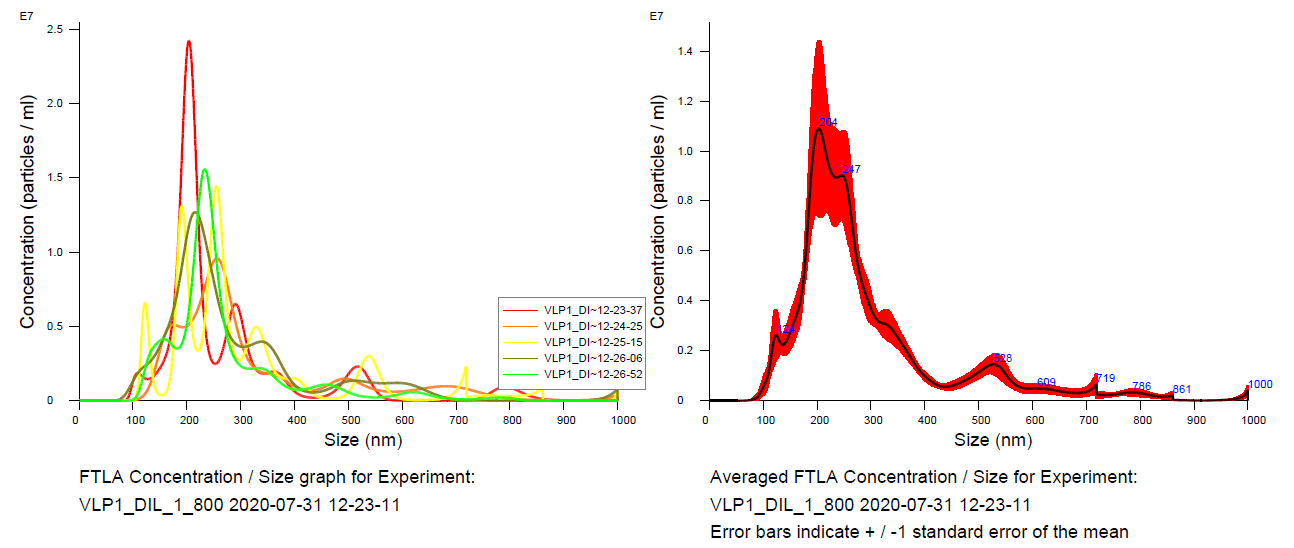


(c)
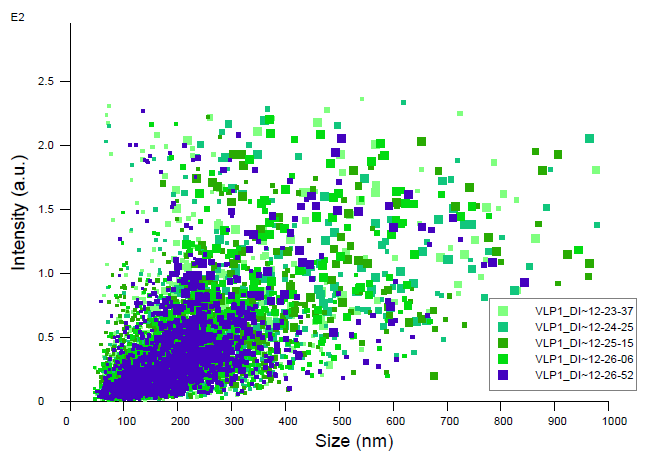


(d)

| **Capture Settings:**  Camera Type: sCMOS  Laser Type: Blue405  Camera Level: 14  Slider Shutter: 1259  Slider Gain: 366  FPS: 25.0  Number of Frames: 749  Temperature: 25.0 ºC  Viscosity: (Water) 0.9 cP  Dilution factor: 1/800 | **Results:**  Stats: Merged Data  Mean: 291.1 nm  Mode: 203.2 nm  SD: 146.0 nm  D10: 169.5 nm  D50: 245.5 nm  D90: 515.4 nm  Stats: Mean +/- Standard Error  Mean: 291.5 +/- 9.8 nm  Mode: 232.6 +/- 10.4 nm  SD: 143.9 +/- 7.9 nm  D10: 169.7 +/- 3.7 nm  D50: 243.7 +/- 8.4 nm  D90: 513.5 +/- 27.2 nm  Concentration: 1.68x10^9^ +/- 7.20x10^7^ particles/ml  85.3 +/- 3.7 particles/frame  145.5 +/- 6.2 centres/frame |
| --- | --- |

**Figure S1.** Characterization of VLPs using a NanoSight Pro, Malvern Panalytical UK, via NTA measurements interpreted with the NanoSight NTA software v3.3.: (a) measurements of size distribution (5 replicates), (b) mean profile of size distribution, (c) comparative 2D plot of the scattered light intensity and size of particles (5 replicates), and (d) report of measurement parameters and results.

| **** | **** |
| --- | --- |
| **** | **** |
| **** | **** |
| **** | **** |

**Figure S2.** Real-time acquisition of raw frequency (black line) in negative value and dissipation data (red line) for a representative sample of COVID-19-positive patients (n_p_ = 25).

| **** | **** |
| --- | --- |
| **** | **** |
| **** | **** |
| **** | **** |

**Figure S2 continuation.** Real-time acquisition of raw frequency (black line) in negative value and dissipation (red line) data for a representative sample of COVID-19-positive patients (n_p_ = 25).

| **** | **** |
| --- | --- |
| **** | **** |
| **** | **** |
| **** | **** |

**Figure S2 continuation.** Real-time acquisition of raw frequency (black line) in negative value and dissipation data (red line) for a representative sample of COVID-19-positive patients (n_p_ = 25).

**Figure S2 continuation.** Real-time acquisition of raw frequency (black line) in negative value and dissipation data (red line) for a representative sample of patients COVID-19-positive (n_p_ = 25).

| **** | **** |
| --- | --- |
| **** | **** |
| **** | **** |
| **** | **** |

**Figure S3.** Real-time acquisition of raw frequency (black line) in negative value and dissipation (red line) data for a representative sample of control subjects (n_c_ = 25).

| **** | **** |
| --- | --- |
| **** |  |
| **** | **** |
| 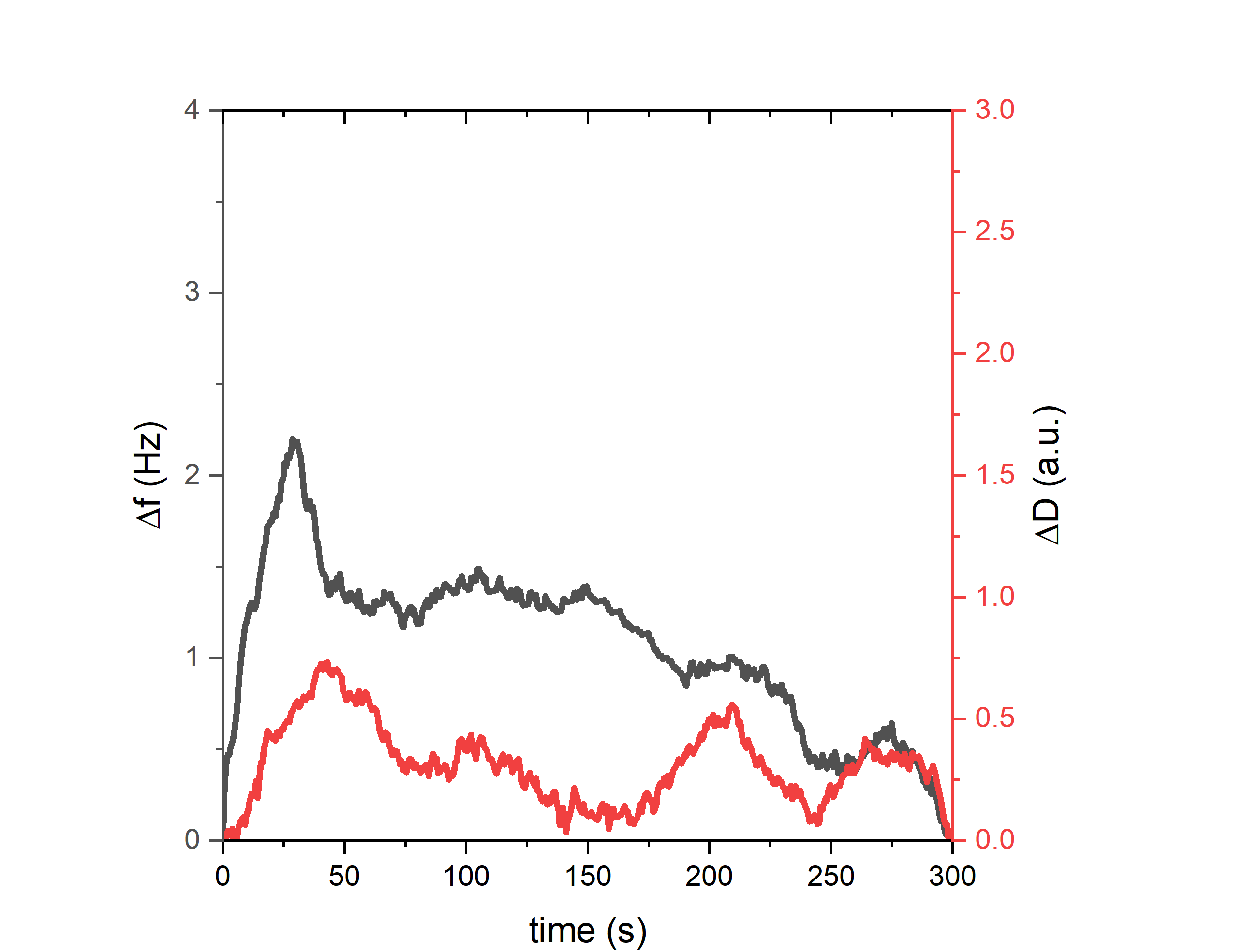 |  |

**Figure S3 continuation.** Real-time acquisition of raw frequency (black line) in negative value and dissipation (red line) data for a representative sample of control subjects (n_c_ = 25).

| **** | **** |
| --- | --- |
| **** | **** |
| **** | **** |
| **** | **** |

**Figure S3 continuation.** Real-time acquisition of raw frequency (black line) in negative value and dissipation (red line) data for a representative sample of control subjects (n_c_ = 25).

**Figure S3 continuation.** Real-time acquisition of raw frequency (black line) in negative value and dissipation (red line) data for a representative sample of control subjects (n_c_ = 25).

| a)  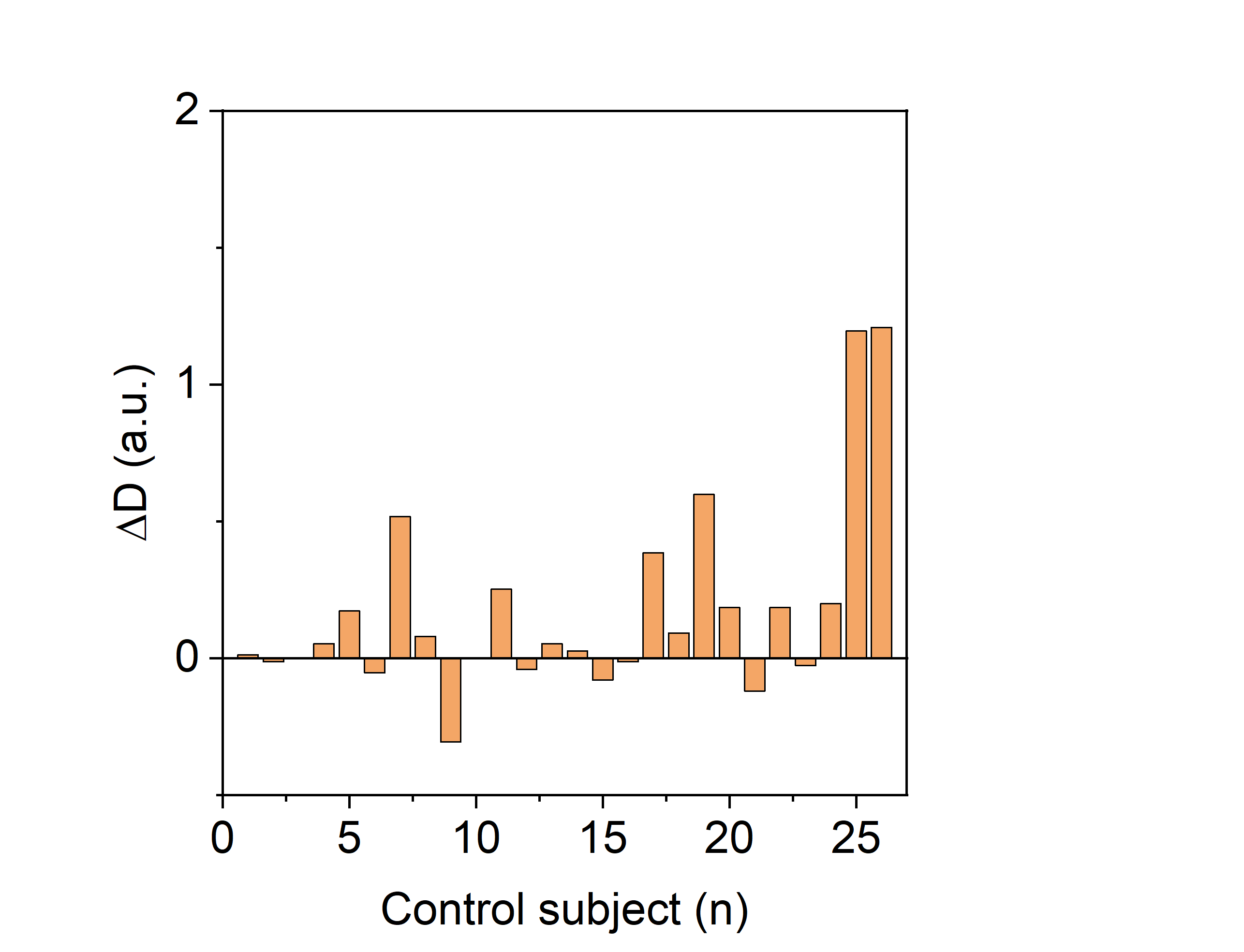 | b)  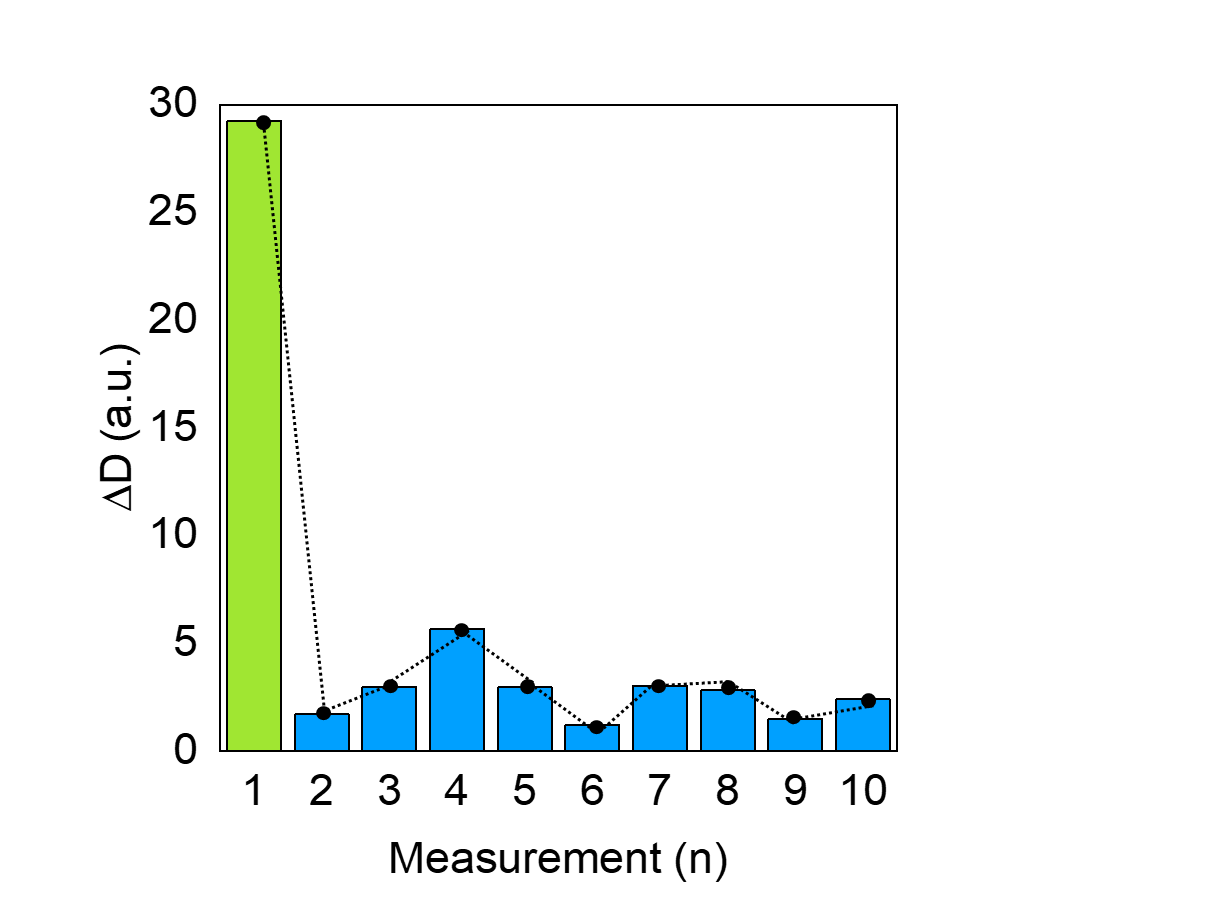 |
| --- | --- |
| ***Figure S4.*** *a) Virusmeter measurements recorded from 26 control subjects using the same chip. b) Virusmeter measurements from a syntomatic patient diagnosed by nasopharyngeal PCR with 29.1 thermal cycles, utilizing the same chip.* | |
